# Supplementary figures and images for: Choice history effects in mice and humans improve reward harvesting efficiency
Source: PLoS Comput Biol. 2021 Oct 4;17(10):e1009452. doi: 10.1371/journal.pcbi.1009452 (PMC8516315; doi:10.1371/journal.pcbi.1009452)

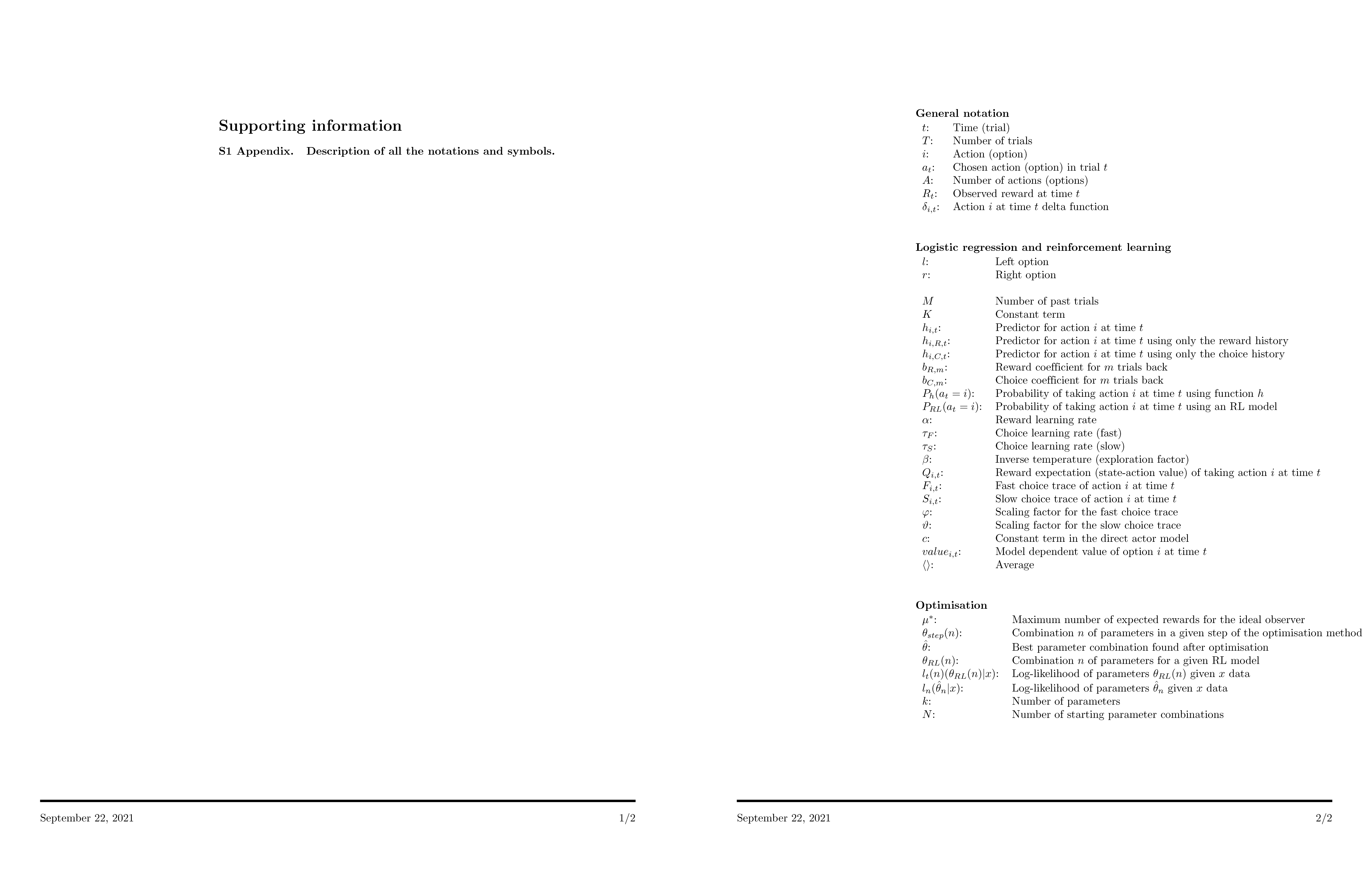

Supplement: S1 Appendix — (TIF) [file pcbi.1009452.s010.tif]
