# Supplementary material for: Choice history effects in mice and humans improve reward harvesting efficiency
Source: PLoS Comput Biol. 2021 Oct 4;17(10):e1009452. doi: 10.1371/journal.pcbi.1009452 (PMC8516315; doi:10.1371/journal.pcbi.1009452)
Supplement: Attachment — Submitted filename: Review.pdf [file pcbi.1009452.s011.pdf]

Lopez-Yepez and colleagues examine the role of choice history in decision making using a binary 2AFC task with a Variable Interval (VI) Reward Schedule; this introduces contingency between the time spent since an option was chosen and likelihood of payout if the option is chosen again. This is different to a lot of 2AFC tasks in which choice biases emerge despite their being no built-in dependency between past choices and current prospects. They find that the choices of animals and humans are influenced by both the history of rewards and history of choices. They also find in mice that the effects of reward history are mediated by volatility (defined as length of the block) whilst effects of choice history are mediated by differences in set reward probabilities. A learning model that incorporates the history of choices is shown to be able to recapitulate these effects, confer advantage over various other models and shown to earn rewards that are closest to maximum possible (indexed via regret) suggesting that incorporating this knowledge helps agents maximise returns.

I like the task and applaud the authors attempt to provide a plausible account of choice biases that are often observed in tasks.

My main set of (related) issues are that in quite a few places, some things about the design and the analysis came across as unclear or confusing.

An example is the experiments the authors run – with mice they run 3 conditions (0.25:0.25, 0.40:0.10 and 0.10:0.40) but with humans they just run 2 conditions (0.40:0.10, and 0.10:0.40). Already it is confusing why this difference in designs exists (no explanation is provided from what I could see). But to confuse things further, in the figure (Figure 2C), it suggests that humans actually did also have the 0.25:0.25 condition? I put a few more examples below. Note – these may each be things that can be tidied up easy enough, but combined it gave me the impression of being a bit careless.

A few other examples (not an exhaustive list):

- Optimal Agents: The analysis of choice history for the 3 optimal agent simulations (Fig. 1) is presented in a haphazard way.
  e.g., why have the number of trials/sessions be different for each combinations of set reward probabilities for the Oracle Agent (P.7)? [For instance, in (1) Number of trials is 1000 and number of sessions is 10 but in (3) Number of trials is either 450 or 1350]
  e.g., why have n=100 sessions for Bayesian agent, n=5000 for the LK agent and n=10 for Oracle?
  e.g., why use a greedy rule for the Oracle but a softmax for the Bayesian and LK Agents?

[Maybe there are good reasons for these differences, but it wasn't obvious to me.]

- Parameter Recovery (Table 1): There are large details missing on how this procedure was carried out. For instance:

how many simulations were run?
why were these specific DT parameter values selected (e.g., are they the average of the parameters fit to the mice data, the human data, both?)
How many trials/blocks were used in the simulations (was it like the human task or the animal task, for instance)
Is it possible to get estimates of variability for the recovered parameters?

- Figure 1B – I think the legend is incorrect as the option with the higher probability (blue line) actually has lower probability of reward for each number of unchosen trials.

Other issues:

- Terminology: The authors describe their approach and task as one that studies "foraging behaviour" (ln97) and explores "foraging decisions" (ln437). Whilst incorporating a nice feature - that time since a specific option was last chosen influences likelihood of its future reward – which is more like some real situations faced by animals outside of the lab, this is definitively *not* a foraging task. The key premise of foraging tasks (such as patch foraging or prey selection) is to have one explicit foreground option that needs to be considered against an estimate of the background reward rate. This is just not the case here; the task used is a 2AFC binary choice task where agents are given an explicit "menu" of all the options available on each trial. See for instance:
    - Hayden, B. Y., & Walton, M. E. (2014). Neuroscience of foraging. *Frontiers in neuroscience*, *8*, 81.
    - Hall-McMaster, S., & Luyckx, F. (2019). Revisiting foraging approaches in neuroscience. *Cognitive, Affective, & Behavioral Neuroscience*, *19*(2), 225-230.
    - Stephens & Krebs, Foraging Theory

- Task (humans): There are only 19 participants in the human task and 20-30 trials per block. This seems very few trials to be able to fit a learning model reliably. Did the authors conduct any checks for this?

- DT Model. It seemed to me a bit peculiar to include both a fast trace and a slow trace of choice history in the model in so far as these quantities have the same update applied to them on every trial. I understand that they end up being different quantities all the same owing to the learning rates being high and low respectively and potentially this enables their model to capture both the fast trial to trial oscillations in the choice effects (e.g., Fig 2c) as well as slower trends over time. Nonetheless, did the authors do any work to untangle whether you need both these traces in their model? (e.g., is one trace doing most of the work in explaining choice history, could a single trace model beat a double trace model?). One option might be to run Model Recovery – simulate data from 3 models (No trace, One Trace, Two Trace) and see which of the 3 models is best fit by the data in each case. For instance, when simulating choices from a model in which there is no trace history incorporated, this should be the winning model (e.g., determined by Bayesian Model Selection) when you fit the 3 contenders to the choices.

- There seem to be some differences in how well the DT model recovers the history effects. For different set probabilities (figure 4a), for the small set probabilities condition, it seems the DT model has a positive effect after a few trials back – but this is not the case in the data (Figure 3b) where effects seems to be asymptote after a few trials at around 0. The size of the effects also seem to differ by a lot. Compare the scale of the y axis on 4a Choice Traces plot (ranges from -1.5 to 0.5) to that of 3B Choice effects (range -4 to 2). Similar differences emerge when you look at the reward history effects for different block lengths – scale of axis in 4B ranges from -0.6 to 0.4, but is in the range -4 to 2 in 3A. Have the authors any idea why these differences arise (it could be something obvious I missed!)?
