# Supplementary material for: Choice history effects in mice and humans improve reward harvesting efficiency
Source: PLoS Comput Biol. 2021 Oct 4;17(10):e1009452. doi: 10.1371/journal.pcbi.1009452 (PMC8516315; doi:10.1371/journal.pcbi.1009452)
Supplement: Attachment — Submitted filename: Response_to_reviewers.docx [file pcbi.1009452.s012.docx]

Dear Dr Kvitsiani,

Thank you very much for submitting your manuscript "Choice history effects in mice and humans improve reward harvesting efficiency" for consideration at PLOS Computational Biology.

As with all papers reviewed by the journal, your manuscript was reviewed by members of the editorial board and by several independent reviewers. In light of the reviews (below this email), we would like to invite the resubmission of a significantly-revised version that takes into account the reviewers' comments.

As you will see, although encouraging, all reviewers point out several issues that should be fully addressed. I would like to attract your attention on the parameter recoverability issue raised by two reviewers. I also would like to add that a model recovery analysis is warranted in your case.

All reviewers pointed out several instances where the paper crucially lacks clarity or non-standard methods are used. We encourage your to better clarify these issues and justify the methods.

We cannot make any decision about publication until we have seen the revised manuscript and your response to the reviewers' comments. Your revised manuscript is also likely to be sent to reviewers for further evaluation.

When you are ready to resubmit, please upload the following:

[1] A letter containing a detailed list of your responses to the review comments and a description of the changes you have made in the manuscript. Please note while forming your response, if your article is accepted, you may have the opportunity to make the peer review history publicly available. The record will include editor decision letters (with reviews) and your responses to reviewer comments. If eligible, we will contact you to opt in or out.

[2] Two versions of the revised manuscript: one with either highlights or tracked changes denoting where the text has been changed; the other a clean version (uploaded as the manuscript file).

Important additional instructions are given below your reviewer comments.

Please prepare and submit your revised manuscript within 60 days. If you anticipate any delay, please let us know the expected resubmission date by replying to this email. Please note that revised manuscripts received after the 60-day due date may require evaluation and peer review similar to newly submitted manuscripts.

Thank you again for your submission. We hope that our editorial process has been constructive so far, and we welcome your feedback at any time. Please don't hesitate to contact us if you have any questions or comments.

**Dear editor**

We have significantly revised the MS and provide requested analysis regarding the parameter recovery. We also carried the new regression analysis that revealed no reward effects on choices. We significantly updated the discussion part in light of the reviewers comments and our new findings.

**Comments to the Authors:
Please note here if the review is uploaded as an attachment.**

Reviewer #1: In this study, the authors investigated the choice history effects in humans and mice during choice behavior reinforced with the variable interval (VI) schedule. They claimed that the observed choice history effects have an advantage in foraging behavior in a natural foraging environment. Given that the choice history effects have attracted growing attention, studies on their adaptive functions are of much interest. But I think there are many issues to be fixed in this paper: There are several places where the methods are not clear, and the validity of the models and analysis is somewhat doubtful. Perhaps this is due to the author's use of their customized method without being fully aware of standard modeling methodologies. I think that the authors should make sufficient efforts to fix these issues. Below are the main issues, especially regarding the methods. But if the authors are willing to revise the manuscript, I recommend that the authors review and revise the entire manuscript.

We thank the reviewer for providing many critical points to address. We have significantly revised the MS and provide more clarity regarding the methods used.


1. Task description

The behavioral task descriptions are insufficient, so the readers cannot obtain information to reproduce the experiments. For example, the paper contains no information about the sex of the subjects (of both mice and humans). How many trials did Mice experienced per session? How long is each session? How did the authors create the program of the computer game for human experiments? Was this done online or in a lab? Why are the trial numbers different across subjects? Also, there is no information on the experimental equipment for mice.

We significantly revised the methods section (see rodent behavioral tasks and human behavioral tasks in methods section, lines 627-666) and provide more details regarding the methods used to perform behavioral experiments.


2. Models

line 571- The Oracle agent
- I could not follow why Eq (7) become looks like this. Where did “(1 – P(R|I,t))(1 - \delta_{I,t})” come from? What exactly does "during block transition" (line 575) mean? If this is an expression that depends on the task design, it should be explained in the task description instead of this section.

We agree with the reviewer and correct this mistake. Eq.7 provides complete description of how probabilities are updated in VI task as it takes into account also the change in set reward probabilities that happens during block transitions. For example when the set reward probabilities changes from one block to another ( i.e. a block with 0.4 vs 0.1 changes into 0.4 vs 0.1) the update eq.7 take into account the change in set reward probabilities. Eq.1 only describes change in probabilities when the set reward probabilities are fixed, thus it provides more narrow definition of how probabilities are updated. We now only provide full description (former Eq.7 is now Eq.1 see in between the lines 129-130) of how probabilities are updated.

line 577- The Bayesian agent
- I'm not sure if the update rule of this model is a calculation that can be called "Bayesian." At first glance, it seems to perform a sampling-based approximation to Bayesian inference, like the sequential Monte Carlo method or the particle filter. However, looking closely, that is not the case. Eq. (12) certainly follows Bayes' formula, but using it for each samples of the parameter does not necessarily lead to that the samples of θ approximate a Bayesian posterior distribution. Also, I cannot figure out how adding a noise factor (line 596) will affect the distribution. If there is research using such a method, the reference is required. If it is the author's original, it is necessary to explain why this update rule should be Bayesian.

The confusion in seeing the update equation as Bayesian may stem from the misspelling. The correct equation should be P(Q_n_(*i*)|R) = P(R|Q_n_(*i*)) * P(Q_n_(*i*)). This is corrected in the revised version of the MS. P(R|Q_n_(*i*)) is the likelihood , and it is a distribution. In other words, it specifies a distribution of probabilities (Q_n_) given that option was rewarded or not rewarded. We also provide additional supplementary (Fig.1 figure suppl.1C) to show that this update correctly infers the set reward probabilities. The reason we add noise factor to the posterior is to avoid the problem that posterior tends to converge on a local minimum, especially when set reward probabilities is very low, 0.1 or very high - 0.9. You can see this effect still in Fig.1 figure suppl.1C.

The reason we get this effect we think is due to the following reason. In baiting structure of the task reward probability on unchosen option increases non linearly. This increase is faster at the beginning and asymptotes for later trials. Also, increase in reward probabilities on unchosen option is more pronounced for higher set reward probabilities (Fig. 1A). This results in assymetric update of the reward probabilities on every trial for unchosen options and the higher is the set reward probability the bigger is this asymmetry. This means that for high set reward probabilities unchosen option’s likelihood quickly asymptotes and as a result posterior settles on lower reward probabilities than true, set reward probability. To overcome this we used a noise term to let the model explore the space, which significantly improved the inference. Overall this results as we understand is similar to approaches used in hierarchical Bayesian models that use noise term in parameter estimation (Mathys C. et al, Front.in Human Neurosc. 2011).

While we find that Bayesian model deserved its own investigation, we thought that the detailed investigation of this model was outside of the scope of this MS.

We provide more clear and detail description of the Bayesian model in Methods section (line 687-693).

line 604- Optimal RL agent (LK model)
- It is unclear to me in what sense this LK model can be called an optimal RL. What does the LK model stand for? I could not figure out why the Qset updated by Eq (16) equals P_set. I could not follow subsequent calculations either.

In the MS Qset is not equal to P_set, it is equal to estimate of P_set, or \hat{P_set}. Now, since the P_set is the set reward probability, we are able to borrow terminology from the RL framework that defines Q as the value of an action in a given state, where the value is defined by the expected sum of returns.

We note that Eq. (15) describes the true reward probabilities, while Eq. (17) computes the estimated reward probabilities. Also, normally \delta is used to describe the prediction error. However, we state the prediction error as PE, and use the \delta to describe a Kronecker delta function (i.e. 1 when action = i, and 0 otherwise). Last, \phi and \alpha are dynamic terms with the purpose of being more adaptive to more general tasks, tasks that span from baiting to non-baiting environments.

LK model is named after first and last author of this MS.

LK model is optimal in the same sense as RL models are optimal if certain environmental contingencies are maintained (i.e., Markovian property). LK model is designed to learn the baiting structure of the environment that none of the RL models aim to capture. We also clarify what exactly LK models aims to learn in the introduction to LK model description in the Methods section (line 706-710). See also our responses below.

line 636 “The learning rate should then be affected by the prediction error of the baiting rate.”
- It is unclear why this can be said and what kind of logic will be used in the subsequent update rule, Eq (20).

Different from standard RL models, LK model performs two approximations concurrently that are interdependent. One approximation attempts to estimate the set reward probabilities, while the other attempts to approximate the “baiting state” of the environment. By combining these two, LK model achieves better than standard RL models, approximation of the actual reward probabilities.

More specifically, the term \phi determine how “baited” is a task, i.e., going from non-baiting (i.e. an armed bandit task) to a baiting task (the VI task). If we suppose that the agent is agnostic about the baiting condition of the task, then it would be useful to have a model that can adapt to either of those tasks dynamically. This term “phi” resets to zero every time the action i is chosen. If \phi is zero, then the agent is assuming the task is not baited, at least in that specific trial. This \phi will increase in unchosen trials only if the prediction error is off the actual value. This would signal to the agent that the task is baited.

line 643- Linear regression for computing reward and choice history effects
- It is difficult to understand whether Eq (21) is an equation (How do the left and right sides correspond? Why the parameter \lambda is included in the left side while not in the right side?). It is also necessary to explain why L1 regularization and L2 regularization are used instead of the standard logistic regression.

Thanks for noting the typo regarding the term lambda. We correct the mistake that previously showed \lambda on the left side of Eq. (21) (see in between the lines 765-766).

The Eq. (21) as it stands now is a standard representation of the penalty terms normally used for regularization of linear regression as described in Zao and Hastie Journal of Royal Soc. 2005. We added this reference in the revised MS (line 761). The regularization is useful when the predictors, the reward and choice histories, show correlations, as it is the case in our data. In VI task autocorrelation in choice history (i.e. alternation) directly effects the reward outcomes. In order to disambiguate effect of past choices and rewards we decided to use regularized linear regression.

Eq (22) is an equation that can be applied to any number of choices, A, but it should be noted that Eq (23) holds when there are only two choices.

That is correct. We have made a correction in the text that Eq. (23) and onwards refers to the two choices case (line 785-788).

lines 670-680
It seems that maximum likelihood estimation is performed with an original method, but why did not the authors use the established and packaged nonlinear optimization method (which can be executed by the function fmincon in Matlab)? Since it seems that the gradient of the objective function has not been estimated, it might take extra time to converge. If there is literature that examines the validity of the method used here, it should be cited.

The method we used follows the basic structure of a Simulated Annealing Algorithm for combinatorial optimization problems (Bertsimas , tsitsiklis simulated annealing, statistical science 1993). It is a set of algorithms with fast convergence, particularly for parallel computation. They are useful for finding global minimum avoiding usually local minima traps. Thus, while the fmincon of Matlab is practical, it is based on continuous gradients for continuous functions and may converge to local minima. Also, the fmincon function, might take longer time to converge than the algorithm we used. Besides, algorithm we use can be considered for more general cases (such as non-convex loss functions) and higher dimensional spaces. We indicate the referenced material in the revised MS (line 800-801).

lines 681-687
Also, I have never seen a document in which AUC is also used in this way. I don't know how to calculate it. I think it needs an explanation. Authors should cite any references available.

AUC stands for area under receiver operating curve, sometimes abbreviated as AUROC. It measures the performance of a binary classifier. We also agree that it is not used very frequently in measuring the performance of the models, but are frequently used in psychophysics-based, two alternative choice tasks to measure performance of animals (Churchland and Carandini. Nat.Neuro. 2013, Green, D.M. & Swets, J.A. Signal Detection Theory and Psychophysics,1966). In our case AUC is the additional metric (besides log likelihood and exceedance probabilities) because it quantifies the performance of the ideal observer. We include the reference in the revised MS (line 325-328).

line 712- Derivation of the DT model from logistic regression
- The DT model consists of a combination of two choice kernels with fast decay and slow decay. I think this can be introduced quite naturally. I'm not sure why it needs to be derived from logistic regression, as the authors discussed. I think it makes sense to associate it with logistic regression for a deeper understanding of the model property, but I'm not sure if the derivation here works for that purpose.

We agree that derivation might not be an adequate word. We simply describe how logistic regression leads to the DT model (line 834).

p.46- RL models
I understand that “Indirect actor” is a natural Q-learning update, but I couldn’t understand how the update rule for the “Direct actor model” (64) was derived. An explanation is necessary.

When there is only one state the actor critic RL model reduces to average reward (R) RL model (Schwartz,1993., Sakai and Fukai, 2008) that is equivalent to direct actor model (Dayan P. and Abbott L., Theoretical Neuroscience). The direct actor model learns the policy directly and thus comes the name as it is described in the Eq.64. We provide a better explanation with references both in the result section (line 300-304) and in methods section (line 934-939), how direct actor model derives its behavior.

p.48
“F-Q up model” is equivalent to the RL model with “default value”, discussed in Toyama, Katahira, & Ohira (2019, Frontiers in Human Neuroscience), albeit this paper assumes that the initial value of Q is set to the default value.

Related to this, the authors should mention how the initial value of Q was set.

We explain in the revised MS how initial values of Q was set (line 951-952).


3. Results

Eq (1) (p.6)
Why is “P_set, i (R)” denoted like a function of R? Isn't this a constant?

We replaced the Eq.(1) with the new version that corrects this notation and also takes into account the block transitions (line 129-130).

line 211 “The discrepancy in choice history effects of optimal agents and animals must stem from the fact that optimal agents can precisely infer the reward probabilities while animals can not.”
- The “discrepancy” mentioned in this sentence is not clear. I think it would be better to show the actual data rather than refer to Lau & Glimcher (2005).

We provide reference to our own data (line 245-248) as shown in Fig. 1C-E and Fig. 2C.

line 233
How the data were split into five parts? Did the authors split sessions within each subject? Or did they ignore the subject identity and pool all the sessions?

We provide a better explanation in the revised MS. We state that “Briefly, the behavioral data (choice and reward histories) were concatenated across all sessions and all subjects and were split into five parts. The model parameters were computed on 4/5 of the data with a 5-fold cross-validation method”, see line (318-320).

line 270
“This finding was also evident when looking at the representative choice dynamics that the DT model, mice, and humans generated (Fig. 2E)."
- I could not figure out how this was evident from Fig.2E.

We simply want to state that DT model is tracking the subject’s choices in a representative session. We swapped the figure 2D with Fig 2E in the revised MS. This way we first show the performance of the DT model on an example session and then compare RL models for the entire data.

Table 1 (p.16)
Only a single simulation of parameter recovery was reported. Parameter recovery should be done many times while changing the true parameters. Please see the following paper:
Wilson, R. C., & Collins, A. G. (2019). Ten simple rules for the computational modeling of behavioral data. elife, 8, e49547.

We performed parameter recovery for 1000 randomly chosen parameters and plot the correlation of true vs recovered parameters. We also did the recovery 200 times for the randomly chosen parameter values. We report this findings in the Fig.2 figure Suppl.3 of the revised MS.

line 380
“Due to the symmetric nature of the two choice traces, whereby different signed parameters can yield the same predictions, we restricted \varphi to negative and \vartheta to positive values.”
- I think this statement is inappropriate. Because \varphi was defined as the weight for *first* decay and \vartheta was defined for *slow* decay, it is not symmetric.

We try to clarify this statement. We agree that it might be confusing the way current MS describes the parameter optimization process. It is true that choice traces are not symmetric, however the same choice history is used to generate both “fast” and “slow” choice traces. Since parameter optimization for \vartheta and \varphi is done repeatedly for different simulations to minimize the regret, nothing constrains the sign of the \vartheta and \varphi as long as they minimize the regret. Thus, we thought it was necessary for the optimization algorithm to maintain the consistent sign for these parameters across different sessions. Besides, animal derived choice traces always showed negative \varphi for fast choice and positive \vartheta for slow choice trace. In the revised MS we clarify these statements (line 451-455).

How did the authors determine model parameters for Figure 4? (Was it selected to minimize the regret?)

Yes, we also indicate this in the revised MS (line 452-453).


4. Other points

Throughout this paper, the authors argue that VIs are more ecologically valid. But I do not think this is always the case. While this may be the case for herbivore foraging, the variable ratio (VR) schedule, in which rewards do not depend on choice history, is more appropriate for carnivore foraging (see Sakai & Fukai, 2008, Neural Computation).

We removed the statement about VI task and its ecological validity.

Also, it would be interesting to analyze the data in terms of whether it follows the matching law. (e.g., Does the choice history effect promote or inhibit matching behavior?) As Sakai & Fukai demonstrated that actor-critic model (but not Q-learning model) can explain the matching law, it may be better to include an actor-critic model as a candidate model.

As we noted earlier (see you comments starting with p.46- RL models) the direct actor model is using the same algorithm to compute the action value as the actor-critic model that uses one state. As Sakai & Fukai showed in their paper actor-critic model requires ~4000 trials to match the rewards with choices when challenged with Variable Interval and Variable Ratio schedules (Fig. 1 of the paper Sakai & Fukai). This happens because actor critic model uses very low learning rate (\varalpha = 0.005) to achieve matching. However, we know that subjects in our VI task can adapt to the block transitions that lasts only ~100 trials. So it would be impossible for the direct actor model to quickly adapt to task contingencies with such a low learning rate. Besides, in all our model comparisons direct actor model was inferior to DT model both in predicting subjects choices and in reward harvesting efficiency in simulated environment. We discuss this topic in the separate section in discussion (“DT model and sequential foraging tasks”) in the revised MS (line 592-614).

Reviewer #2: General comments:
Junior Samuel Lopez-Yepez and his colleagues are interested in the issues of choice history effects. The authors are interested in the “decoupling” of reward and choices in the sequence of trials. In the natural habitat, the choices act on the environment and change its state, which subsequently influences the reward richness of the environment. But such inter-trial dependency of the choices and rewards are not carefully examined in the previous studies. They devised the task paradigm with a variable interval (VI) reward schedule, which incorporates such inter-trial dependencies of choices and rewards. By examining the behaviors of humans and mice on this task paradigm, the authors have found that application of the double trace (DT) model, which was originally applied to the reward history effects of monkeys, to choice history effects can explain the behavior of animals well. Therefore, the value of the paper depends on how the VI task paradigm is novel and in introducing the intertrial reward-choice dependencies and on how the DT model is adequate in describing and explaining the interactions of choice and reward in long-term scales beyond a single trial. Overall, there is some puzzling aspect in this paper. The conclusion of the paper is just about the choice history effects but the task design and the initial aim of the paper is so explicitly set out to examine the effects of the long-term contingency of choice and rewards. The authors need to address the disconnections in the design, analysis, and the interpretation in this study.

We thank the reviewer for raising the critical issue with choice and reward dependencies. Our revised MS addressed this issue and we include a new figure and discuss its implications.

Major points:


1. The first issue is how much of this new task paradigm is effective for elucidating the effects of long-term choice-reward contingency compared to the previous paradigms. As the authors mentioned, the task paradigms inspired by foraging theory has uncovered interesting patterns of the long-term history effects of choice and reward (Hayden et al., Nature Neuroscience, 2011; Kolling et al., Science, 2012; Kolling et al., Neuron, 2014; Wittmann et al., Nature Communications, 2016; Wittmann et al., Nature Communications, 2020). The authors should conduct more careful comparisons of these existing literatures.

We have included in the discussion part a new section. “DT model and sequential foraging tasks” that addresses the raised issue (line 592). In short all these papers except of Hayden et al. use different flavours of average reward RL models to explain subjects choices which is analogous to direct-actor model (see our response to reviewer1 above). In Hayden et al choices are also determined by long running reward average and while authors do not use average reward RL, the Marginal Value Theorem assumes that agents know this average. So overall, in mentioned papers animals rely one way or another on average reward rate. What separates our behavioral paradigm and DT model from work in mentioned papers is that we don’t see any effect of average reward rate on choices. Briefly, these are the reasons why we think long range dependencies between global reward rates and choices is not holding up in VI task. 1. New regression analysis as suggested in comment3 failed to reveal any effect of past rewards (unsigned rewards or rewards unlinked to choices) on choices. 2. When we manipulated reward rates in animals (Fig. 3E) we failed to see any effects on choice or reward history effects. 3. Direct-actor model that is analogous to average reward RL models was worse at predicting subjects choices and was also inferior than the DT model in reward harvesting efficiency (Fig.4 A,B).

2. Related to the first point, the previous literature of foraging decision paradigms with long-term contingency of choice and reward elucidated mainly the history effects of rewards in the previous trials (Wittmann et al., 2016; Wittmann et al., 2020). In contrast, the authors claim that the long-term history effects are mainly due to the choice history effects. It is crucial to reconcile the difference of these interpretations between the current results and the previous results using the conceptually similar tasks.

Maybe we were not clear in the MS , but we don’t want to claim that long term history effects are mainly due to previous choices and rewards don’t play a significant role. We only provide behavioral function to choice history effects. While we agree that our task and tasks published by (Wittmann et al., 2016; Wittmann et al., 2020) are using probabilistic structure of the environment, there is also a significant difference. Namely in VI task reward probabilities on unchosen options grow while in published work reward rates on unchosen options are static. This introduces a different contingencies for subjects and requires different behavioral policies to maximize their reward collection. We are discussing these issue in the revised MS (line 602-607)	.

3. Separation of the choice history effects and reward history effects in the behavioral analyses are basically artificial in the current study and in the previous studies. This issue is more pronounced in this study because authors explicitly aimed to examine the effects of the across-trial contingency between the choices and rewards. The analyses in this study, however, linearly separated history effects of choices and rewards. It might be more straightforward to examine such “interaction terms” of choices and rewards if the authors are genuinely interested in these intricate relationships between choices and rewards.

We agree that a better way to decipher the past reward and choice effects would be to perform regression analysis as suggested using interaction terms. We report in Fig. 2 figure Suppl. 2 the new regression analysis (also see “extended linear regression model” in Methods section). We note that past rewards (unlinked to choices) have no effect on current choices. However, we discover that no rewards have negative effects on choices that explains first negative coefficient of choice history effects on choices. This new findings we report in the result section (line 252-267) and discuss it’s implications (line 576-580). Briefly, we think that choice history effects combine effect of no rewards and past choices. In light of this finding, what DT model achieves is to parametrize effects of rewards, no rewards and choices into reward and choice traces.

4. DT model of “choice history” effects might contain the similar problem of the artificial separation of the choice and rewards. The author’s focus on the choice history effects is understandable given the recent popularity of the issue of the choice history effects or decision inertia. But it is also puzzling that the conclusion of the paper is just about the choice history effects given that the task design and the initial aim of the paper is so explicitly set out to examine the effects of the long-term contingency of choice and rewards.

Our revised analysis as described in previous response addresses this issue. With the discovery of no reward effects we think that choice history effects are partly explained by reward history. We describe this in Fig.2 figure Suppl. 2. The slow trace of the choice history effects on the other hand is difficult to explain with any reward contingencies as we failed to see any effect of choice-unlinked rewards on choices (see our responses to previous comment).

Minor points

1. The article uses concepts or variables (alternation, perseverance, volatility) without introducing them first, which is pretty confusing, even if those variables can be more or less intuitive. The results section did not give enough information for the analyzed variables to be intuitive enough. I needed to go back and forth from the results to the methods constantly, and sometimes to find that the methods did not contain the information I was looking for either.

We define in the results section what we mean by alternation (line 181-182), perseverance (line 182-183) and volatility (line 367-368).

2. Figures are also incomplete and not everything that appears is labeled or explained. The text gets particularly confusing when dealing with the particular dataset each analysis was based on. Some were based on simulations (optimal agents or their proposed model), while others are based on mouse and human data. In the case of simulated agents, there are several reward distributions tested for optimal agents, and it looks like sometimes block transitions are used and sometimes not. In the case of biological data, two mouse datasets are used, and most of the analyses are not done with the human dataset at all. The authors should make an effort to clarify the particularities of the environment data was simulated/collected on, and to justify why each kind of analysis was done on only certain datasets.

We have worked on each figure to explain everything that is labelled. We are adding a new statement why we used different task conditions in synthetic agents (line 153-154). We also explain in the text that the last task condition used for the Oracle agent is the one that was used for the remaining agents and one that is closest to the task conditions used for humans and mice (line 165-166). The reason we used no block transitions for the Oracle agent was to estimate its performance on a uniform probability distribution that is impossible to do in animals (line 185-189) that turned out to be later similar to the animal generated choice history effects. The reason we did not use same exact task for humans is that we were limited by the number of trials that humans could perform. Different from water deprived mice humans have less incentives to perform boring task. So not to be confounded by attentional and motivational mechanisms we severely limited the number of trials in humans. This also applies to why we could not use the same task as we used for mice (Fig.3) in humans as it would require the same subjects to run the same version of the task for thousands of trials. We provide justification in the revised MS (line 234-237).

3. In the authors summary (page 3), authors make clear what process their model captures which other models do not. Namely, and in their own words, the fact that “animals track their history of visits and use it to maximize the food harvesting efficiency". To me that makes a lot of sense. However, it is difficult to find this clear message in the main text, particularly in the discussion.

We changed both introduction and discussion and tried to simplify many terms.

Specific points

PAGE 3
• Lines 55-57: the authors suggest that “choice history bias” is synonymous with “choice inertia”, while that is not exactly true. Inertia is a particular kind of bias where the probability of repeating a choice increases the more a choice is repeated (Akaishi et al., Neuron, 2014).

We do not use term inertia in the text in the revised MS, but would like to cite the paper (Akaishi et al., Neuron, 2014) when explaining the DT model’s choice trace (line 277-278).

PAGE 4
• Lines 71-72: I find the way the (or initial) probability in the VI task is explained a bit confusing. Maybe it is because it starts by saying “in each trial rewards are assigned with fixed or set reward probabilities keyed to different options”, and it is not until much later that it is said that the probability also depends on the number of elapsed trials without that option being chosen. Maybe authors should start by saying that each option’s reward probability is defined by both a set (or initial) probability, unique for each option, and the number of trials without that option being chosen.

We agree that rephrasing the sentence that describes probabilities may convey more clear message (Line 77-80).

PAGE 5
• Lines 89-90: The affirmation “To maximize their reward rate, optimal agents should choose the options with the highest set reward probability” assumes that both options lead to a reward of the same magnitude, if this is delivered (e.g. 1 point). Maybe this should be stated before when describing the VI task, as many decision-making studies implement different rewards for each option.

We indicate that in VI task equally sized rewards are assigned to different options (Line 72-73).

• Lines 92-93: The authors say that an optimal strategy in this environment may “resemble choosing the best option, with occasional switches to lesser options”. This statement is not well connected to the remaining part of the paragraph, but it could make an interesting point to be developed in the discussion. It would be particularly interesting to connect it to what previous work thinks such lapses mean (i.e. <https://elifesciences.org/articles/55490>).

We moved that sentence in the discussion part and discuss it within a context of lapses in perceptual decision-making tasks (line 556-562).

PAGE 8
• Lines 171-173: The peaks observed in simulated data should be discussed a little bit.

We provide explanations why peaks appear in the optimal agents choice history effects (Line 193-201).

PAGE 16
Lines 303-304: a short definition of volatility should be introduced here, as it is important to understand the following analyses. The authors need to explain what “reward history effects are sensitive to the volatility” means. In the original paper, it was learning rate, which was affected by the volatility (Behrens et al., Nature Neuroscience, 2007).

We explain what we mean by volatility (line 367-369).

PAGE 20
• Figure 3: the figure and the legend need serious reworking. Why are the middle panels on the left hand side of A, B and C needed? They simply depend on experimenters’ fixed parameters. What do yellow dots mean? What kind of variability are we being shown in each panel with those error bars? What do red crosses mean? What do blue and grey circles represent on the right hand side panels? Also it should be said that the volatility analyses were made on a different dataset than the rest of the analyses described in the figure, for which new data (although with the same participants) was gathered.

We agree that parameters that were set by experimenter can be simply reported in the text i.e. difference in set reward probabilities and block length, but rewards rates depend on the performance of the animal, so we kept it in the figure. Yellow dots indicated parameters that were manipulated, but in the updated figure they don’t exist anymore. Each panel shows boxplot with median and 25^th^ and 75^th^ percentiles, whiskers show the extreme data points that algorithm (MATLAB function boxplot) did not consider as outliers and red crosses as outliers. Blue and grey circles are also not present any more in the figures. We only state that we compare first regression coefficients using Pearson correlation analysis ( we indicated that information in the Fig.3 legend of the updated MS). We also indicate that the behavioral manipulations were done on a new set of animals (line 362-365).

PAGE 22
• Lines 410-411: why do authors think that the DT model’s choice trace reflects volatility, but they do not find that in data?

We think that the DT model’s choice trace as shown in Fig.4 might be noisy and not reflect the task contingencies fully. Our parameter recovery showed that (Fig. 2 figure Supll. 3) the parameters for choice trace are particularly noisy, while parameter recovery for reward trace (learning rate show good approximation of the true learning rate) are more precise. We indicate this issue in the revised MS (line 481-483).

• Lines 414-436: the first paragraph of the discussion is way too long and it does not summarize the achievements of the present work. It only gives context, and it would be more appropriate for the introduction.

We moved that paragraph in introduction section.

PAGE 23
• Lines 424-432: the authors take a convoluted path to make a simple point, which, if I understand correctly, is the following. Some experimental work whose primary aim is studying optimal behavior, but not necessarily within the domain of perception, still resorts to perceptual tasks. But assessing optimality in perceptual tasks has extra nuisances that we could disregard in simpler, non-perceptual scenarios, such as the one proposed here. I feel like this point could be made easier.

As suggested in previous comment we moved that part in the introduction and simplified the message. We no longer discuss the issue with perceptual decision making tasks.

• Line 435: what property are the authors talking about?

We meant the fact that in two alternative choice tasks everything else being equal the cost of different choices are the same. In a simplified version, this message should be more clear (Line 97-105).

PAGE 24
• Lines 442-444: authors say that “optimal agents show the same characteristic shape of choice history effects observed in animals when probed with uniform distribution of set reward probabilities”. I think this is quite confusing. The key information to understand this is to bear in mind that the optimal agents the authors are talking about have access to the exact set probabilities of each option. Thus, in this case their “knowledge” transparently reflects the experimental parameters. Maybe authors want to make that clear.

In the revised MS we remove that statement.

• Lines 445-447: I do not see how point #2 falls from what the authors have previously reasoned in that paragraph.

We also removed this statement in revised MS.

PAGE 25
• Lines 480-483: I feel this is an overstatement. Just because these models take reward history into account it does not mean they will offer an accurate description of behavior and neural activity.

We restated the sentence in the revised MS (line 543-547).

• Line 483: why the “however”? We remove word however.

PAGE 26
• Lines 502-515: this is a paragraph about neural representations, and yet no neuroimaging work is cited.

We cite human fMRI work that describes the neural correlates of multiple time scales of learning (line 588-590).

• Line 507: sometimes authors spell “time scales”, sometimes “time-scales”. A unified spelling should be used.

We corrected that mistake.

PAGE 27
• Lines 516-534: Charnov’s article aside, this paragraph only has one citation. Authors should try to cite more sources.

We cite more references (line 598).

• Line 536-540: the conclusion should succinctly summarize the most important points of the manuscript, yet the authors use it to suggest, for the first time, that choice history effects are phylogenetically conserved. Maybe this evolution-related bit should be moved to the discussion, and the conclusion should focus on a short wrap-up.

We rewrote completely the conclusion and are focusing on key findings (line 615-625).

Reviewer #3: Lopez-Yepez and colleagues examine the role of choice history in decision making using a binary 2AFC task with a Variable Interval (VI) Reward Schedule; this introduces contingency between the time spent since an option was chosen and likelihood of payout if the option is chosen again. This is different to a lot of 2AFC tasks in which choice biases emerge despite their being no built-in dependency between past choices and current prospects. They find that the choices of animals and humans are influenced by both the history of rewards and history of choices. They also find in mice that the effects of reward history are mediated by volatility (defined as length of the block) whilst effects of choice history are mediated by differences in set reward probabilities. A learning model that incorporates the history of choices is shown to be able to recapitulate these effects, confer advantage over various other models and shown to earn rewards that are closest to maximum possible (indexed via regret) suggesting that incorporating this knowledge helps agents maximise returns.

I like the task and applaud the authors attempt to provide a plausible account of choice biases that are often observed in tasks.

We thank the reviewer for pointing to parameter recovery issue.

My main set of (related) issues are that in quite a few places, some things about the design and the analysis came across as unclear or confusing.

An example is the experiments the authors run – with mice they run 3 conditions (0.25:0.25, 0.40:0.10 and 0.10:0.40) but with humans they just run 2 conditions (0.40:0.10, and 0.10:0.40). Already it is confusing why this difference in designs exists (no explanation is provided from what I could see). But to confuse things further, in the figure (Figure 2C), it suggests that humans actually did also have the 0.25:0.25 condition? I put a few more examples below. Note – these may each be things that can be tidied up easy enough, but combined it gave me the impression of being a bit careless.

The reason we used fewer conditions in humans (also explained in response to reviewer 2 comment 2, above) was that we were limited by the number of trials we could run for humans as the task is fairly boring and we did not want to be compromised by the attentional and motivational mechanisms. So this was the reason why we also did not test human performance for contingencies as described in Fig.3. We correct also the mistake on Fig.2C. We lay out these reasons in the revised MS (line 234-237).

A few other examples (not an exhaustive list):
• Optimal Agents: The analysis of choice history for the 3 optimal agent simulations (Fig. 1) is presented in a haphazard way.
e.g., why have the number of trials/sessions be different for each combinations of set reward probabilities for the Oracle Agent (P.7)? [For instance, in (1) Number of trials is 1000 and number of sessions is 10 but in (3) Number of trials is either 450 or 1350]
e.g., why have n=100 sessions for Bayesian agent, n=5000 for the LK agent and n=10 for Oracle?
e.g., why use a greedy rule for the Oracle but a softmax for the Bayesian and LK Agents?

The reasons we used different trial length for different agents are also described in our response to reviewer 2 , comment 2 (see above). The reason we used different action selection rule for Oracle and other agent is because the Oracle knows the exact probabilities and does not explore options (thus greedy algorithm). While the softmax is used for agents that need to explore the environment to converge to the best solution. We make it clear in the revised MS (line 172-176).

[Maybe there are good reasons for these differences, but it wasn’t obvious to me.]

• Parameter Recovery (Table 1): There are large details missing on how this procedure was carried out. For instance:
how many simulations were run?
why were these specific DT parameter values selected (e.g., are they the average of the parameters fit to the mice data, the human data, both?)
How many trials/blocks were used in the simulations (was it like the human task or the animal task, for instance)
Is it possible to get estimates of variability for the recovered parameters?

This point was also raised by the first reviewer. We have run simulations using the same block length (100 trials) that falls within the range of block length defined for mice (50-150 trials) in VI task. For the probabilities we used 0.1 vs 0.4, 0.4 vs 0.1 probabilities for left vs right options. We carried a new analysis for parameter recovery and report these finding in Fig. 2 figure Suppl. 3.

• Figure 1B – I think the legend is incorrect as the option with the higher probability (blue line) actually has lower probability of reward for each number of unchosen trials.

Yes, it is true we mislabeled the lines. We correct it in the updated MS.

Other issues:

• Terminology: The authors describe their approach and task as one that studies “foraging behaviour” (ln97) and explores “foraging decisions” (ln437). Whilst incorporating a nice feature - that time since a specific option was last chosen influences likelihood of its future reward – which is more like some real situations faced by animals outside of the lab, this is definitively not a foraging task. The key premise of foraging tasks (such as patch foraging or prey selection) is to have one explicit foreground option that needs to be considered against an estimate of the background reward rate. This is just not the case here; the task used is a 2AFC binary choice task where agents are given an explicit “menu” of all the options available on each trial. See for instance:
o Hayden, B. Y., & Walton, M. E. (2014). Neuroscience of foraging. Frontiers in neuroscience, 8, 81.
o Hall-McMaster, S., & Luyckx, F. (2019). Revisiting foraging approaches in neuroscience. Cognitive, Affective, & Behavioral Neuroscience, 19(2), 225-230.
o Stephens & Krebs, Foraging Theory

It depends how one defines foraging. If foraging is defined as comparing available option (foreground) with the background reward rate than our task is not a foraging task. In VI task as we described in our response to reviewer 2 comment, average reward rate RL model does not provide adequate predictive power and subjects do not take into decision rule average reward rate. In that sense, our task is not a foraging task. However, in our understanding foraging simply meant searching for better alternatives. Animals in searching for food may use different rules than comparing average reward rate with available options. Yet, to be consistent with the previous work we note this distinction in the discussion part. We are extensively discussing this part in paragraph “DT model and sequential foraging tasks”.

• Task (humans): There are only 19 participants in the human task and 20-30 trials per block. This seems very few trials to be able to fit a learning model reliably. Did the authors conduct any checks for this?

We limited the number of trials in humans as not to be compromised by the attentional mechanisms in a fairly boring task. Our DT model generated good prediction accuracy more than 70 % of correct trials on a test data.

• DT Model. It seemed to me a bit peculiar to include both a fast trace and a slow trace of choice history in the model in so far as these quantities have the same update applied to them on every trial. I understand that they end up being different quantities all the same owing to the learning rates being high and low respectively and potentially this enables their model to capture both the fast trial to trial oscillations in the choice effects (e.g., Fig 2c) as well as slower trends over time. Nonetheless, did the authors do any work to untangle whether you need both these traces in their model? (e.g., is one trace doing most of the work in explaining choice history, could a single trace model beat a double trace model?). One option might be to run Model Recovery – simulate data from 3 models (No trace, One Trace, Two Trace) and see which of the 3 models is best fit by the data in each case. For instance, when simulating choices from a model in which there is no trace history incorporated, this should be the winning model (e.g., determined by Bayesian Model Selection) when you fit the 3 contenders to the choices.

What we did is to compare in model comparision test (Fig. 2D), no choice trace model (F-Q model), single choice trace model (F-Q W/C ) and DT model. We show that DT model is superior in model selection (Fig.2D) as well as in reward harvesting efficiency (Fig.4). This would argue in favour of including two choice trace in the RL model. We further tested (as suggested) how different models would match DT model generated data. As we show in the revised MS (Fig.2D), DT model generated data is best recovered by the DT model itself.

• There seem to be some differences in how well the DT model recovers the history effects. For different set probabilities (figure 4a), for the small set probabilities condition, it seems the DT model has a positive effect after a few trials back – but this is not the case in the data (Figure 3b) where effects seems to be asymptote after a few trials at around 0. The size of the effects also seem to differ by a lot. Compare the scale of the y axis on 4a Choice Traces plot (ranges from -1.5 to 0.5) to that of 3B Choice effects (range -4 to 2). Similar differences emerge when you look at the reward history effects for different block lengths – scale of axis in 4B ranges from -0.6 to 0.4, but is in the range -4 to 2 in 3A. Have the authors any idea why these differences arise (it could be something obvious I missed!)?

The DT model choice and reward traces in Fig. 4B,D are generated by parameter recovery, that can be noisy, especially for choice trace (Fig. 2-figure Suppl. 3), while in Fig. 3 we use logistic regression with regularization to estimate the choice and reward history effects. Also note that the Oracle agent’s choice history effects (Fig. 1C left panel for choice history effects) are close in the range to the animal generated data. We note in the revised MS that inconsistencies between the optimized DT model and animal generated choice trace can be explained by noisy recovery of the \vartheta and \varphi parameters (line 481-483).
