# Supplementary material for: Choice history effects in mice and humans improve reward harvesting efficiency
Source: PLoS Comput Biol. 2021 Oct 4;17(10):e1009452. doi: 10.1371/journal.pcbi.1009452 (PMC8516315; doi:10.1371/journal.pcbi.1009452)
Supplement: Attachment — Submitted filename: Response_to_Reviewers.docx [file pcbi.1009452.s013.docx]

**Comments to the Authors and Authors responses:**

Reviewer #1: I am grateful to the authors for responding to my previous suggestions. However, I am still concerned about “Bayesian agent.” In addition, there is an additional issue regarding the significance of this study, which I noticed after I wrote the first review report. Since I was unable to point this out during the initial peer review, I would like to leave it to the editor to decide whether or not to take this into account.


Regarding the Bayesian agent:

In response to my previous comments regarding the Bayesian agent, the authors responded as: “The confusion in seeing the update equation as Bayesian may stem from the misspelling. The correct equation should be P(Qn(i)|R) = P(R|Qn(i)) * P(Qn(i)). We also provide additional supplementary (Fig.1 figure suppl.1C) to show that this update correctly infers the set reward probabilities.”

However, this is not enough as an answer. If one calls a Bayesian agent, the agent should represent the distribution of the variable of interest (in this case, the set reward probability). What Fig.1 suppl.1C shows are the point estimate of set reward probability rather than its distribution (By the way, the title of figure suppl.1C includes misspelling). Representing the posterior distribution requires at least making sure that its variance (uncertainty) can be expressed according to the Bayes formula. In the authors’ framework, the distribution of the estimates for the set reward probability, \theta^n(i), should obey the posterior distribution. However, the authors did not evaluate this. When we are interested only in point estimator, we may call it Bayesian estimation if it provides the maximum a posteriori (MAP) estimator. However, it is unclear whether this is the case (whether the parameter corresponds to the MAP estimator rather than the maximum likelihood estimator) in the author's model.

Also, regarding adding the noise, the authors wrote as:
“Overall this results as we understand is similar to approaches used in hierarchical Bayesian models that use noise term in parameter estimation (Mathys C. et al, Front.in Human Neurosc. 2011).”
As far as I understand, the Mathys et al. approach assumed noise in the generation process, but the estimation is done deterministically under variational approximation, which is quite different from adding noise to the estimates as the authors did.

The Bayesian inference model is a concept that is becoming well established in behavioral modeling. Calling the author's model a Bayesian agent may cause confusion. If the author cannot properly construct a Bayesian inference model, then the Bayesian agent model should be removed from the manuscript, or the name should be changed.

***Our response****: We change the name for the Bayesian agent and simply refer to this as inference based (IB) agent that uses elements of the Bayessian update rule (line 138-139).*


Additional issue regarding the significance of the paper:

I noticed that Houston and McNamara (1981, Journal of the experimental analysis of behavior, 35(3), 367-396), which was also cited in the manuscript, theoretically showed that the periodic deterministic choice behavior gives the highest reward in the concurrent VI schedule. In addition, periodic (alternation) choice strategy have been commonly observed in VI-schedules without change over delay (COD) procedure, which seems absent in the authors’ experiment, as stated as follows in Corrado et al. (Corrado, G. S., Sugrue, L. P., Seung, H. S., & Newsome, W. T. (2005). Journal of the experimental analysis of behavior, 84(3), 581-617.):

“A second feature that our foraging task shares with many classical matching paradigms is the incorporation of a changeover delay (COD). The COD is a common technique for introducing a ‘‘cost,’’ in this case a temporal delay, to switching from one choice option to another (Shahan & Lattal, 1998). Thus, although graded matching behavior can be observed without the use of a COD (see, e.g., Lau & Glimcher, 2005), its incorporation shields the data from partial contamination with competing behavioral strategies based on alternation. Without such a cost, an animal can gather rewards surprisingly efficiently by alternating between options…”

Thus, I think that it is already known that alternating behavior, which appeared as the negative history effect of the previous trial observed in the authors' results, maximizes reward under certain VI schedule conditions. Given all this, what new knowledge can we say that the results of this paper bring? I would like to see a discussion of COD and the author's contribution in light of these previous studies.

***Our response****: To be clear, the periodic choice strategy described in Houston and McNamara’s (1981, Journal of the Experimental Analysis of Behavior, 35(3), 367–396) paper diverges from the short-term alternation that we and others (Lau and Glimcher 2005) see in experimental data. For example, the agent may choose the left option for four consecutive trials and switch to the right option only for one trial. This behavior would be periodic but would not be qualified as short-term alternation (switching between right and left options for every trial). This paper reveals that deterministic behavior with a fixed number of choices for the rich option should be followed by a single choice for the leaner option. The number of consecutive choices for the rich option depends on the difference in the interval of reinforcement in the case of a continuous VI schedule or the difference in set reward probabilities in the case of a discrete version of the VI task. Short-term alternation is the optimal strategy only if the two options are very similar in terms of reinforcement interval or reinforcement set probabilities.*

*Corrado, Sugrue, Seung, and Newsome (2005) mention that alternation (likely short-term) can be surprisingly efficient. However, they do not clarify what they mean by efficiency, that is, efficiency compared to what strategy? Corrado and colleagues refer to Stubbs, Pliskoff, and Reid (1977), who claim nothing about the efficiency of the alternation strategy in a discrete version of the VI task. N.b. discrete and continuous versions of the VI task can give rise to disparate optimal strategies (Houston & McNamara, 1981, Journal of the Experimental Analysis of Behavior, 35(3), 367–396).*

T*o settle the argument, we can compare the short-term alternation strategy in the VI task (with set reward probabilities of 0.1 vs. 0.4) and depict this alternation is worse than at least 53 other strategies. If an agent selects a 0.4 probability option in consecutive trials (two to 54 consecutive trials) and a 0.1 probability option only once (see the plot below), this strategy will result in better performance than alternation. Of course, an agent can choose a low-probability option all the time, but it is trivial that this agent does not maximize the reward rates. In the case of 0.25 vs. 0.25 probability cases, short-term alternation is indeed the best strategy. Although short-term alternation is not optimal (unless set reward probabilities are the same for the two options), we still see strong short-term alternation in Oracle agents, as shown in Figure 1C. We conclude that this alternation emerges in Oracle agents only when set reward probabilities are drawn from a uniform probability distribution. Thus, short-term alternation per se does not maximize the reward rate; it maximizes the reward rates only when the environment provides a uniform reward probability distribution.*

Average reward rate (y-axis) as a function of consecutive trials (x axis) for high probability option (0.4) and switch for one trial to the low probability option (0.1).

*Furthermore, it is not that animals simply alternate for every trial; otherwise, their regression coefficients for past choices would be only negative for the immediately prior trial back and zero for the remaining previous trials (Figure 2C). Instead, choice history effects are slightly positive for two or more trials. Animals use short-term alternation and long-term perseverance, the same strategy in Oracle agents when facing uniform probability distributions.*

*To our knowledge, these findings have never been reported in previous papers.*

*Finally, we also show that choice history effects (especially short-term alternation) can be tuned to the statistics of the environment (Figure 3) and are not fixed behavioral patterns. The previous studies, to our knowledge, the previous studies also did not show behavioral contingencies that drive choice history effects in VI tasks.*

*Overall, what our MS brings to the field is that choice history effects (with short-term alternation and long-term perseverance) emerge in animals as a reward maximization strategy in habitats where exact reward probabilities are hidden and animals assume uniform reward probability distribution.*

*We thank the reviewer for bringing up this issue with alternation. We are happy to extend our discussion on this topic to the revised MS (line 507-520).*

Minor points:

Line 282:
“The higher the inverse temperature, the more stochastic the choices are…”
- I think this is the opposite.

***Our response****: Thanks for bringing this up. We correct the mistake (line 284).*

Line 357:
were significantly different (p = 0.0006,…
- “were significantly different from zero” or “were significant”?

***Our response****: Here we compare choice ad reward history first regression coefficients and report how different they are. We have rephrased the sentence to make it more clear (line 359).*

Line 446
“,Here an agent takes action a duringin each trial t.”
- The sentence is broken.

***Our response****: We correct the misspelling (line 450).*

Reviewer #2: Authors has addressed the previous comments sufficiently. The manuscript improved with the borader perspective on this issue. Because the previous manuscript was so unfocused and confusing with the methodology, I could not see some central points of the paper. With the further analyses in this version, there are some new finding that is relevant the central claims of the paper such as the finding of the effect of the no-reward for the immediately preceding choice. So I would like to ask the authors to address the following points:

1. Can we still call the model a "double" trace model of choice history effects given that the fast component is based on no-reward on the previous choice?

***Our response****: As long as we explain what behavioral processes double trace captures, we would prefer to keep the term “double trace” as it is.*

2. Choice history effect as rational or irrational bias depends on its relevance to the task. Clearly in this task the elapsed time (trials and number of choices on the options) is directly connected to the possible reward on another option. In contrast,  in other studies such as Akaishi et al. (2014), Fritsche et al. (2017). and Akrami et al.(2018), the choice history bias is irrelevant to the task performance. I am just wondering whether the interpretation of the choice history bias in the current task may not generalize to other tasks with the task irrelevance of the biases.

***Our response****: The reviewer has raised an interesting point, which is somewhat related to the issue raised by Reviewer 1 regarding the significance of the MS. Strictly speaking, with tested set reward probabilities (0.4 vs. 0.1, 0.1 vs. 0.4, 0.25 vs. 0.25), the choice history effects that we observe in animals are not maximizing reward rates. As one can see from Figure 1 C–E, when optimal agents are tested with the same set of reward probabilities, choice history effects are quite different from what we observe in animals (Figure 2C). The reward maximization function of choice history effects is clear only when the Oracle agent is tested in a task with a uniform distribution of set reward probabilities. Therefore, we argue that choice history bias can be seen as serving the reward maximization function only under certain assumptions of the reward probability distribution. Thus, in the narrow perspective, the choice history bias in our study can also be considered irrational or irrelevant to the task structure. Yet, seen from a broader view of how animals infer the probabilistic structure of the environment, choice history effects may maximize reward rates.*

*Nonetheless, the distinction between our task and those in cited papers is clear. In our task, we impose a contingency between previous and current choices and manipulate those contingencies, while in other tasks, contingencies are not explicitly manipulated. We discuss the implications of these issues in the updated MS (line 570-578).*

3. This is relatively minor point but this necessary for good scholarship with sufficient documentation of previous literature. The neural bases of the multiscale representation of the past choices have been reported. For example, single unit activities in the prefrontal cortex have been found to be related  with the fast and slow choice biases.

Mochol, Kiani, Moreno-Bote
Current Biology, 2021Prefrontal cortex represents heuristics that shape choice bias and its integration into future behavior
<https://doi.org/10.1016/j.cub.2021.01.068>

One of the earliest studies of choice history bias in the past decade (Akaishi et a., 2014, Neuron), from which authors burrowed the equation of the double trace model, has reported the extensive evidence about the neural bases of the multiscale choice history bias. The study described not only the involvement of the prefrontal but also the medial parietal areas and its interaction with the prefrontal cortex.

***Our response****: We thank the reviewer for providing the overlooked references. We update the MS accordingly (line 602-608).*

Reviewer #3: my comments have all been addressed
