# Supplementary material for: Choice history effects in mice and humans improve reward harvesting efficiency
Source: PLoS Comput Biol. 2021 Oct 4;17(10):e1009452. doi: 10.1371/journal.pcbi.1009452 (PMC8516315; doi:10.1371/journal.pcbi.1009452)
Supplement: Attachment — Submitted filename: Response_to_Reviewers.docx [file pcbi.1009452.s014.docx]

**Comments to the Authors and Authors responses:**

Reviewer #1: I think that the authors addressed my comments. I am now happy to recommend publication.

Reviewer #2: The authors responded to the questions of the reviewers partially in the new submission of this manuscript. In this resubmission, the authors added a critical piece of the new data regarding the "double trace model of choice history effect". In the previous round of the review, I have asked the authors the justifications of the use of the  "double trace model of choice history effect". Below I reproduce the interaction:

My comment: With the further analyses in this version, there are some new findings that are relevant to the central claims of the paper such as the finding of the effect of the no-reward for the immediately preceding choice. So I would like to ask the authors to address the following points:

Major point 1. Can we still call the model a "double" trace model of choice history effects given that the fast component is based on no-reward on the previous choice?

Author's response: As long as we explain what behavioral processes double trace captures, we would prefer to keep the term “double trace” as it is. 

My point was: if the alternative models incorporate both history of reward-choice interaction and history of choices, which is the implication of the new results, can we still call it the "double trace model of choice history effect"? There are confusions of the term "double trace model" throughout the paper regarding the components of the "double trace model". If it is purely consisting of choice history effects, please clearly mention this. And if the author means this, the  issue posed by the new results of "the effect of the no-reward for the immediately preceding choice" has to be resolved.

Usually the authors need to answer the questions of the reviewers not with the "preference" but "justification". It is a long review process but it is the responsibility of the authors to keep a good manner of interactions.

We apologize if the reviewer felt his comments were ignored. Overall we strongly believe that all the comments and criticism have substantially improved the MS and we thank all the reviewers for their work.

The confusion we believe stems from the term “choice history effects” that in the light of the new data combines immediate past no reward and more distant past choice effects. However, this data has to be interpreted with caution. Just because mathematically choice history effects can be decomposed into no reward and past choice effects it does not mean that biologically they are driven by the same mechanisms. It is entirely possible that some part of the brain (habitual system like basal ganglia circuits) implements choice history effects by relying only on past choices (ignoring no rewards and rewards) and combines it with reward history effects (computed in other parts of the brain) as described by the DT model. However when analyzed with the regression model (Fig.2-figure Suppl. 2), it will look like as if animals respond to no rewards and past choices. In such a case negative first coefficient for past choices will reduce positive reward first coefficient for past trials on rewarded trials. On non rewarded trials only negative past choice coefficient will remain. Likewise one could explain the rest of the coefficients, but this would be misleading characterization of the behavior. Currently we have no way to disambiguate these two possibilities. One way to reveal this mechanism is to probe the neural circuit mechanisms that generate them. In a hypothetical experiment imagine one inactivates pure choice system (as postulated by the DT model) leaving reward system intact. This inactivation should increase choices to previously rewarded options, but if one inactivates only no reward system the same choices to rewarded options should not change. Given this ambiguity in interpreting the choice history effects without proper experimental tests, we preferred to stick to the original version of the definition of the choice trace. In the updated MS we indicate that these are two alternative ways one could interpret choice history effects (line 255-271, also see in the discussion, line 514-518).

We clarify in the updated MS what behavioral variables two choice traces (*F* and *S*) in the double-trace model correspond (line 280-281). The reason we think it is justified to keep the name of the model as double trace that refers to the choice history effects are 1. The choice history effects (or choice kernels) as a name was used in previous papers (Lau glimcher, Kim), hence it made sense for us to use the same definition when incorporating it in the DT model 2. Oracle agent (Fig.1C second left panel) when faced with uniform probability distribution generates very similar choice history effects as animals do (Fig.2C). While Oracle agent does not respond to rewards or no rewards, its past choices have very similar effects on current choices. It could be a coincidence that these choice history effects resemble each other or it could be driven by the same mechanisms. Again we have no way to prove it, but this in our opinion also justified to keep the name.

The editor in the previous round of the review asked the authors to clarify the novelty of the paper given that VI task has been used in the past studies. The authors need to address this point sufficiently as well.

Different from previous studies that used VI task structure, 1. It remained unclear what behavioral contingencies drive choice history effects. 2. Biological function of choice history effects was not tested. Our behavioral manipulations revealed that growth of reward probabilities on unchosen option drives choice history effects. Modeling work revealed that choice history effects help animals to maximize the reward harvesting efficiency. We note these points in the updated MS (line 496-503).

Also, within the discussion, in the main paragraph of page 27 the authors say "Indeed, in many decision-making tasks that do not impose contingencies of previous choices on current choices, choice history effects persist (Akaishi et al., 2014; Fritsche et al., 2017; Akrami et al., 2018; Hwang et al., 2017; Mochol, Kiani, & Moreno-Bote, 2021). Understanding the behavioral mechanisms that generate choice-history effects in these tasks may require the manipulation of behavioral contingencies, linking past choices to current choices in a parametric way." Authors can mention the papers that discuss how experimental instructions may make changes of choice history effects, or if there is any review comparing the size of choice history effects across different paradigms (foraging tasks with "replenishing" patches vs tasks with static reward probabilities, for instance). In the paradigms of perceptual decisions, (trial) frequencies of each stimulus condition in a block can bias the decisions (and possibly choice history effects too) such as the following study:

Elapsed Decision Time Affects the Weighting of Prior Probability in a Perceptual Decision Taskhttps://www.jneurosci.org/content/31/17/6339.short 

The comparison to these papers clarify the value of the current paper in the background related studies.

We indicate papers (including one cited by the reviewer) that reveal the adaptive role of bias or autocorrelations in task-relevant stimuli in forming choice history effects (line 596- 599). We further note that in contrast to perceptual decision tasks (to our knowledge) in reward foraging tasks no behavioral manipulation has been performed to reveal the function of choice history effects. However, theoretical work (Possingham H.P. and Houston A. 1990 , J.Theor.Biol.) shows that for patch foraging agents the optimal strategy should take into account the replenishing rates of unvisited patches. Supported by experimental work (Hall-McMaster et all 2021 iScience) manipulation of replenishing rates on different unvisited patches results in more frequent visits to fast replenishing sites as well as early leaving times from those sites in human subjects. While these studies did not look at the choice history effects these finding are consistent with our data that behavior of animal (choice history effects) is sensitive to growth rate of probabilities on unchosen options. We cite these paper in the updated MS (line 599-610).

Some minor/technical points

It is still not clear why the authors conclude that participants assume uniform distributions of set reward probabilities. In the current version of the manuscript, the end of the first paragraph of page 12 refers the reader to two figures. But these figures are not that self-explanatory to me. I think a short explanation in that paragraph would help. Particularly because that is brought up again in the discussion, and the information there is not sufficient either.

We explain the figures both in the main text as well as in the figure legends. We also update the labels of the supplementary figure (line 243-254, legend for Supplementary figure 1)
